# Personal

**Published:** 1891-12

**Authors:** 


### Personal.

---

DR. ERNEST WENDE, of Buffalo, has been appointed Health Commissioner of this city, under the new charter, which takes effect January 1, 1892. It is understood that Dr. Wende was not an applicant for the place himself, and accepts it upon the urgent solicitation of friends, at great personal sacrifice. It is one of the few instances where the office has sought the man, and is an evidence of progress, for it is difficult to see how it could have made a better choice. Dr. Wende is the leading dermatologist of Western New York, and brings an admirable equipment in education and personal fitness to his new field. We hope he will enjoy his official work, but hardly understand how he can afford to abandon his large special practice for even so tempting an office.

---

DR. W. E. B. DAVIS, of Birmingham, Ala., was elected president of the Tri-States Medical Association at the recent meeting, held in Chattanooga, Tenn. Dr. Davis is an accomplished physician and an experienced presiding officer,—two good reasons why it may be expected that his administration of the affairs of the Association will prove successful in the highest degree.

---

PROF. W. T. BRIGGS, his many friends will be glad to learn, has fully recovered from his recent severe illness, and is able to attend to business again.—*Nashville Jour. of Med. and Surg.*

Dr. C. A. L. Reed read a paper at the eighty-fifth annual meeting of the Medical Society of the State of New York, upon The Surgical Treatment of Ectopic Gestation. The article was published in the *New York Medical Journal*, of June 6, 1891. The paper is full of good points, and can safely be regarded as a real contribution to our knowledge upon this subject. We advise all who are interested in the subject to read this article and judge for themselves.—*Lancet-Clinic.*

---

Dr. John A. Pettit, of Buffalo, has been appointed Deputy Health Commissioner under the new charter. Health Commissioner Wende shows his fitness for his new position in this first official act. Since Dr. Pettit is entirely competent for the chief position, there cannot be two opinions as to his ability to perform the duties of deputy in a thorough-going manner. When the head of a department selects able and honest men for subordinate places, his successful administration is more than half assured.

---

Dr. Wm. A. Wheeler, United States Marine Hospital Surgeon, formerly of this city, and now of Norfolk, Virginia, visited Buffalo last month, and received a most cordial welcome among his many friends. Dr. Wheeler justly deserves the reputation of being one of the most accomplished surgeons in the service, and we join in the high estimation in which he is held both here and elsewhere in and out of the service.

